# Mitigation of the Interplay Effects of Combining 4D Robust With Layer Repainting Techniques in Proton-Based SBRT for Patients With Early-Stage Non-small Cell Lung Cancer

**DOI:** 10.3389/fonc.2020.574605

**Published:** 2020-10-09

**Authors:** Long Wei, Haijiao Shang, Fu Jin, Yuenan Wang

**Affiliations:** ^1^School of Computer Science and Technology, Shandong Jianzhu University, Jinan, China; ^2^Shanghai Institute of Applied Physics, Chinese Academy of Sciences, Shanghai, China; ^3^University of Chinese Academy of Sciences, Beijing, China; ^4^RaySearch, Shanghai, China; ^5^Department of Radiation Oncology, Chongqing University Cancer Hospital, Chongqing Cancer Hospital, Chongqing Cancer Institute, Chongqing, China; ^6^Department of Radiation Oncology, National Cancer Center, National Clinical Research Center for Cancer, Cancer Hospital & Shenzhen Hospital, Chinese Academy of Medical Sciences, Peking Union Medical College, Shenzhen, China

**Keywords:** lung cancer, proton SBRT, 4D robust optimization, interplay effects, layer repainting

## Abstract

**Objective:**

The objective of this study was to evaluate the interplay effects in proton-based stereotactic body radiotherapy (SBRT) using 4D robust optimization combined with iso-energy layer repainting techniques for non-small cell lung cancer (NSCLC).

**Materials and Methods:**

Twelve patients with early-stage NSCLC who underwent 4DCT were retrospectively selected. A robust CTV-based 4D plan was generated for each based on commercial Treatment planning system (TPS), considering patient setup errors, range uncertainties, and organ motion. The 4D static dose (4DSD) and 4D dynamic dose (4DDD) were calculated using a hybrid deformable algorithm and simulated proton delivery system. An index $\Delta {\text{I}}_{M}^{R}\left(\%\right)$ was developed to quantitatively evaluate the interplay effects. The interplay effects of the 4D robust plan and multiple iso-energy layers (3, 4, 5, 6, and 7) of the robust repainting 4D plan were calculated based on $\Delta {\text{I}}_{M}^{R}\left(\%\right)$ to select the optimal times for layer repainting.

**Results:**

Due to the interplay effects, the mean target values D_2_ and D_5_ increased to 1.28 and 1.01%, and the target values D_98_ and D_95_ decreased to 2.01 and 1.77%, respectively, for the 4D Robust SBRT plan. After multiple iso-energy repainting times, the interplay effects of the target values D_98_ and D_95_ tended to migrate, from 2.01 to 0.92% in target value D_98_ and from 1.77 to 0.89% in target value D_95_, respectively. Moreover, a positive linear correlation was observed between the optimal interplay effect mitigation and target range of motion.

**Conclusion:**

In proton-based 4D Robust SBRT, the interplay effects degraded the target dose distribution but were mitigated using iso-energy layer repainting techniques. However, there was no significant correlation between the number of repainting layers and improvements in the dose distributions. The optimal layer repainting times based on the interplay effect index were ascertained according to the patient characteristics.

## Introduction

Proton spot scanning-based stereotactic body radiotherapy (SBRT) has been shown to outperform photon-based SBRT in patients with early-stage non-small cell lung cancer (NSCLC). Proton has a superior Bragg peak increasing the dose delivered to tumors and sparing healthy organs, such as the lungs, esophagus, and spinal cord (1, 2). However, patient setup errors and range and target motion uncertainties must be addressed to make full use of the advantages of proton-based SBRT for lung cancer (3).

The motion of lung tumors has been evaluated based on 4DCT imaging. Most lung tumors have limited motion, less than 5 mm, especially in locally advanced NSCLC (4). Patient setup and range uncertainties are the primary factors to consider during IMPT planning. In early-stage NSCLC, approximately 50% of lung tumors move more than 5 mm and some even move more than 2 cm in the superior–inferior direction (5), where interplay effects caused by the interference between the beam spot and intra-fractional respiratory motion is dominant and should be taken into account. Otherwise, the quality of the dose distribution can be severely degraded. The effects should be minimized as much as possible. Robust optimization combining with 4DCT imaging (4DRP) (6, 7) mitigates the interplay effects. The appropriate repainting strategy (8, 9) can also manage the interplay effects. However, there are few clinical reports on the effectiveness of combining 4DRP and repainting with proton-based SBRT for IMPT in patients with early-stage NSCLC. Moreover, an interplay index for quantitative evaluation is needed to assess the effectiveness of combining 4DRP and repainting. The interplay effects can be estimated by calculating the single-fraction 4D static dose (4DSD) and single-fraction 4D dynamic dose (4DDD) based on 4DCT images (10, 11). The 4DSD is calculated based on the assumption that the tumor moves in 4DCT images without considering the delivery system’s time dependence. The 4DDD is calculated by taking into account the delivery system’s time dependence. However, this method is not very intuitive and cannot provide changes in the target volume or organs at risk (OARs), such as target conformity, homogeneity, and OAR volume dosimetry.

In the current study, a 4D Robust plan was generated for selected patients. The interplay effects were quantitatively evaluated using a target index and OAR index. A multiple iso-energy layer repainting strategy was also used to further mitigate the interplay effects to explore the optimal mitigation outcomes.

## Materials and Methods

### Patient Characteristics

A total of 12 patients with early-stage (IA/IB) NSCLC were selected for this study, which was approved by the local institutional research review board. The clinical target volumes (CTVs) were contoured by the attending radiation oncologists at each 4DCT phase. The internal target volumes (ITVs) were created by encompassing the extent of 10 CTV motions in 10 4DCT phases. The patients’ information is summarized in Table 1. The inclusion criteria were as follows: (i) the tumor was small with no distant metastasis, each <5 cm in diameter, (ii) the patients’ body surface was more than 4 cm away from the tumor and did not require the use of a range shifter, and (iii) the patients underwent specialized respiratory training to maintain a stable respiratory cycle for approximately 3–5 s.

**TABLE 1:** Summary of patient characteristics including tumor location, size, and motion range.

Patient No.	Diagnosis	CTV Volume (Mean ± SD)	Breathing Period(s)	Motion Range (DVF) (cm)
1	NSCLC/IB	40.1 ± 1.67 (cc)	4.2	0.75
2	NSCLC/IA	20.4 ± 1.35 (cc)	4.5	1.02
3	NSCLC/IA	10.5 ± 0.61 (cc)	3	1.12
4	NSCLC/IA	10.3 ± 0.65 (cc)	3.5	1.62
5	NSCLC/IB	22.3 ± 0.71 (cc)	3.8	1.08
6	NSCLC/IA	23.2 ± 1.56 (cc)	3.6	1.27
7	NSCLC/IA	20.9 ± 1.18 (cc)	3.8	0.84
8	NSCLC/IA	21.7 ± 0.56 (cc)	4.2	0.57
9	NSCLC/IA	28.3 ± 0.56 (cc)	4.8	0.68
10	NSCLC/IA	13.8 ± 1.75 (cc)	3.9	1.35
11	NSCLC/IA	20.3 ± 0.66 (cc)	4.8	0.68
12	NSCLC/IB	36.4 ± 1.25 (cc)	4.5	0.75
Median (Range)		21.3 (10.3–40.1)	4.05	0.93

### Target Range of Motion

The target range of motion was obtained by calculating the maximum deformation vector lengths (DVLs) in the target area (9, 12). The maximum inhale phase T_0_ and maximum exhale phase T_50_ were used for deformable image registration (DIR) (13) to obtain the DVL. The DIR algorithm was developed by RaySearch and performs well in lung applications (14). Voxels in the CTVs were selected and the target range of motion was calculated according to the DVL formula:

$$D\,V\,L_{i}=\sqrt{{\left(x_{T_{0,i}}-x_{T_{50,i}}\right)}^{2}+{\left(y_{T_{0,i}}-y_{T_{50,i}}\right)}^{2}+{\left(z_{T_{0,i}}-z_{T_{50,i}}\right)}^{2}}$$

where *x*_T__0,*i*_−*x*_T__50,*i*_,*y*_T__0,*i*_−*y*_T__50,*i*i_,*z*_T__0,*i*_−*z*_T__50,*i*_ are the components in voxel *i* of the deformation vector field between the T_0_ and T_50_ phase images in the 4DCT images.

### 4D Robust Treatment Planning

#### Spot Scanning SBRT

Pencil beam scanning proton plans were generated for the patients via RayStation (RaySearch Laboratories, Version 6.1 sp1, Stockholm, Sweden) using proton energies between 70 and 225 MeV with beam data from a typical pencil beam scanning dedicated nozzle manufactured by IBA (Ion Beam Applications S.A., Louvain-la-Neuve, Belgium). The spot sizes at the iso-center in air varied between 2.5 mm at 225 MeV and 6.8 mm at 70 MeV. At least greater than 95% of the CTV received a prescribed dose of 60 Gy [RBE] in five fractions. Two or three suitable oblique coplanar beams were used for the plan with the beam direction according to the tumor location. Then, a 4D Robust optimization algorithm (7) was used based on the 4DCT images considering the patient setup, range uncertainties, and target motion. Before optimization, the minimum and maximum spot weights were 0.02 and 4 MU, respectively.

#### Uncertainty Modeling

Proton-based SRBT is sensitive to the patient setup, range uncertainties, and organ motion, so all of the uncertainties should be considered in the model. Inter-fractional patient setup uncertainties were simulated by shifting the patient iso-center in the antero–posterior (A–P), superior–inferior (S–I), and right–left (R–L) directions by 5 mm, yielding six dose distributions and the corresponding influence matrices (the beamlet dose distributions per unit intensity). Range uncertainties were simulated by scaling the stopping power ratios by ±3.5% to generate two additional dose distributions and influence matrices corresponding to the minimum and maximum proton ranges, respectively. The organ motion uncertainty was considered using 4DCT images consisting of 10 respiratory cycle phases to generate 10 dose distributions with each respiratory cycle phase.

#### 4D Robust Optimization

The 4D Robust optimization plans (4DRP) were generated by optimizing the CTV dose in 10 4DCT phases considering the modeling uncertainties. Robust optimization taking into account the set S of scenarios was implemented using minimax optimization (15). The objective function was

$$\min_{x\in X}\,m\,a\,x_{s\in S}\,\sum_{i=1}^{n}w_{i}\,f_{i}\,\left(d\,\left(x;s\right)\right)$$

where X is the set of feasible variables (spot weights for spot scanning IMPT), *d*(*x*;*s*) is the dose distribution as a function of variable *x*, scenario *s*, and *f*_*i*_is the *i* structure’s penalty function. The robust optimization objective were used in CTV with a minimum dose objective of 60 Gy [RBE] (weight 100) and a maximum dose objective of 60 Gy [RBE] (weight 60). A dose fall-off function from 60 Gy [RBE] to 10 Gy [RBE] over 1 cm (weight 10) was used to lower the dose to the normal tissue as much as possible. The equivalent uniform dose (EUD) (16) on the normal lung was approximately 5 Gy [RBE] with a dose volume effect parameter of 1 (*A* = 1). In the final dose calculation, the Monte Carlo dose engine was used with 0.5% statistical uncertainty and a 3 mm× 3 mm× 3 mm dose grid resolution.

### Interplay Effect Calculation

**FIGURE 1:**
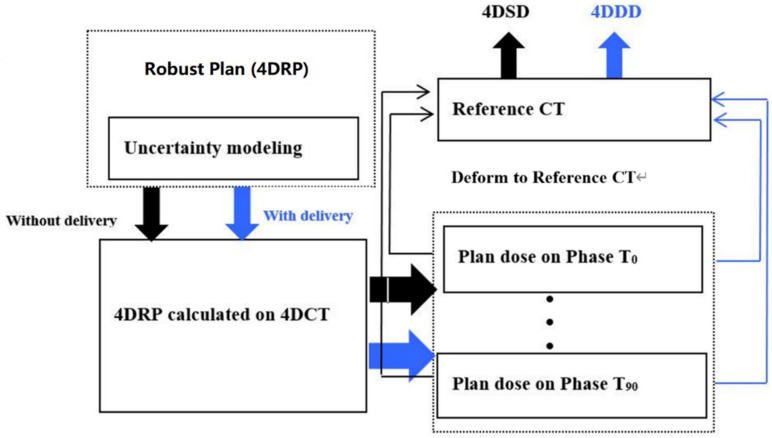
Flowchart of the 4D static dose (4DSD) and 4D dynamical Dose (4DDD) for 4D robustly SBRT plan.

An overview of the interplay effect process is shown in Figure 1. The black flowchart shows how the 4DSD was produced, while the blue flowchart shows how the 4DDD was produced.

The quantitative interplay effect index was represented by the differences in the DVH metrics $\left(\Delta \,{\text{I}}_{M}^{R}\right)$ between 4DSD and 4DDD over 4DSD for each region of interest (ROI).

$$\Delta {\text{I}}_{M}^{R}\left(\%\right)=\frac{4\,\text{DDD}\,\left[{\text{DVH}}_{M}^{R}\right]-4\,\text{DSD}\,\left[{\text{DVH}}_{M}^{R}\right]}{4\,\text{DSD}\,\left[{\text{DVH}}_{M}^{R}\right]}\times 100\%$$

where $4\,\text{DSD}\,\left[{\text{DVH}}_{M}^{R}\right]$ and $4\,\text{DDD}\,\left[{\text{DVH}}_{M}^{R}\right]$ are specific DVH metrics for one ROI in the 4DSD distribution or 4DDD distribution. $4\,\text{DSD}\,\left[{\text{D}}_{95\%}^{\text{CTV}}\right]$ represents the 4DSD at 95% of the CTV volume. If the value of $\Delta \,{\text{I}}_{M}^{R}$ was positive, the DVH metrics increased in the 4DDD distribution and vice versa.

We used the CTV and lung minus ITV (lung – ITV) as the ROIs to obtain the quantitative interplay effects. The DVH metrics in the CTV included the minimum target dose (D_98_[cGy(RBE)]: dose at 98% of the target volume), prescription dose (D_95_[cGy(RBE)]: dose at 95% of the target volume), and maximum dose (D_2_[cGy(RBE)]: dose at 2% of the target volume). The target EUD (A = 10) metrics were also included for interplay evaluation. For lung – ITV, the DVH metrics of the lungs included V_5_, V_20_, and V_30_, which were the percentage volume of lungs receiving 500 cGy [RBE], 2000 cGy [RBE], and 3000 cGy [RBE], respectively. The lung – ITV average dose and EUD (*A* = 1.2) (17) were also calculated for evaluation.

### Iso-Energy Layer Repainting

The repainting strategy used for the 4D robust plans was layered repainting, where each energy layer is rescanned several times before switching to the next energy level. Zenklusen et al. (8) proposed two different methods to divide a plan into layers: scaled and iso-layered repainting. Scaled repainting involves simply dividing each layer in a present number of layers. In contrast, in iso-layered repainting, the MU per spot is limited by the maximum value. In this study, we segmented 4DRP by scaling the repainting 3, 4, 5, 6, or 7 times and obtained new repainting plans called 4DRP-SN3, 4DRP-SN4, 4DRP-SN5, 4DRP-SN6, and 4DRP-SN7, respectively. The layers were divided with respect to the minimum MU per spot, which means that some spots with the same energy levels consecutively contained fewer and fewer spots, but the minimum number of spots was not smaller than the limited minimum MU per spot. For the current study, the machine’s minimum MU (0.02 MU) was used during the division to ensure that all of the spots in the rescans were directly deliverable.

The optimal interplay effect mitigation in the 12 patients was assessed by comparing the value of interplay effect index $\Delta \,{\text{I}}_{D_{98}}^{\text{CTV}}$ and $\Delta \,{\text{I}}_{D_{95}}^{\text{CTV}}$ to obtain the minimum index of $\Delta \,{\text{I}}_{D_{98}}^{\text{CTV}}$ and $\Delta \,{\text{I}}_{D_{95}}^{\text{CTV}}$ over five different iso-energy repainting plans. In other words, the smaller the interplay effect index, the better the interplay effect mitigation outcome.

### Statistical Analysis

Student’s *t*-test was used to compare the following results between 4DRP(**SN3**), 4DRP(**SN4**), 4DRP(**SN5**), 4DRP(**SN6**), and 4DRP(**SN7**), respectively: (1) the interplay effect in the target DVH metrics (D_2_, D_5_, D_95_, D_98_, and Target EUD) and (2) the interplay effect in lung minus ITV DVH metrics (V_5_, V_20_, V_30_, and EUD); *p* < 0.05 was considered statistically significant. We compared (1) and (2) to investigate whether increasing the number repainting layers mitigated the interplay effects in the target and lungs. A linear regression model was used to evaluate the correlation between the optimal interplay mitigation vs. the tumor range of motion. This study was conducted using a linear model created with Excel software version (v.2016, Microsoft, Redmond, WA, United States)^1^ to predict the correction.

## Results

### Interplay Effects of the 4DRP

**FIGURE 2:**
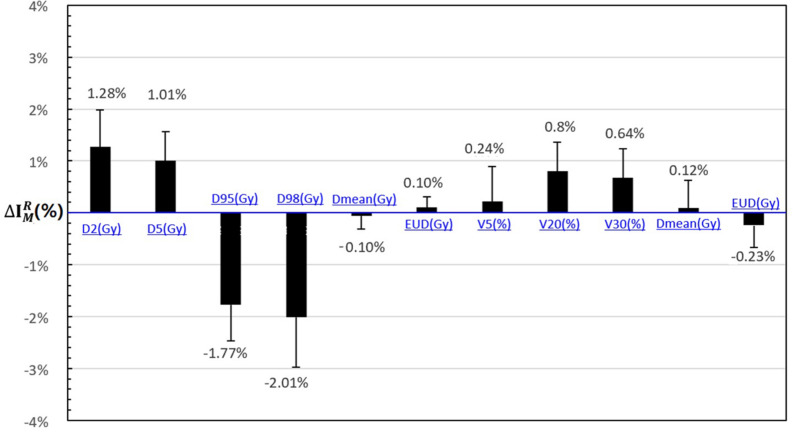
The statistical difference with DVH metrics of interplay index between 4D static dose (4DSD) and 4D dynamical dose (4DDD) for all the patients.

As shown in Figure 2, the DVH target and OAR metrics changed in the 4D robust plan due to the interplay effects. Targets D_2_ and D_5_ increased while D_98_ and D_95_ obviously decreased.

The mean value of $\Delta \,{\text{I}}_{D_{2}}^{\text{CTV}}$ and $\Delta \,{\text{I}}_{D_{5}}^{\text{CTV}}$ increased to 1.28 and 1.01% according to the interplay index, and $\Delta \,{\text{I}}_{D_{98}}^{\text{CTV}}$ and $\Delta \,{\text{I}}_{D_{95}}^{\text{CTV}}$ decreased to 2.01 and 1.77%, respectively. For the OARs, the mean value of $\Delta \,{\text{I}}_{V_{5}}^{\text{Lung}}$, $\Delta \,{\text{I}}_{V_{20}}^{\text{Lung}}$, and $\Delta \,{\text{I}}_{V_{30}}^{\text{Lung}}$ increased less than 1%.

### Interplay Effects After Repainting

**FIGURE 3:**
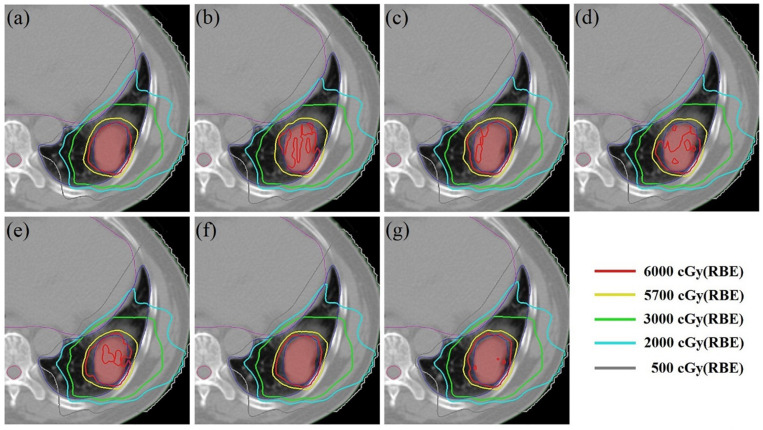
The 4D static dose distribution 4DSD **(a)** and 4D dynamical dose distribution 4DDD **(b)** in the transverse plane for patient #2 of the 4D robust plan 4DRP and 4DDD in the five numbers of layer repainting plan based on 4DRP, marked as 4DRP(SN3) **(c)**, 4DRP(SN4) **(d)**, 4DRP(SN5) **(e)**, 4DRP(SN6) **(f)**, and 4DRP(SN7) **(g)**. The target CTV is red filled and PD line is 6000 cGy (Red), shown as the legend from the dose line.

Using patient #2’s treatment plan as an example, the 4DSD distribution, 4DDD distribution of the 4DRP, and 4DDD distributions of the 4DRP(**SN3**), 4DRP(**SN4**), 4DRP(**SN5**), 4DRP(**SN6**), and 4DRP(**SN7**) were investigated. In the 4DSD distribution, the isodose line of the prescription dose (PD) basically covered the tumor volume (Figure 3a). Considering the interplay effects, the distribution of the dose lines markedly deteriorated, and the PD isodose lines failed to cover the target area (Figure 3b). Further executing the repainting at different layer repainting times, the target area was again covered by the isodose lines of the PD dose lines (Figures 3c–g). No significant changes occurred in the isodose lines of the lungs.

**FIGURE 4:**
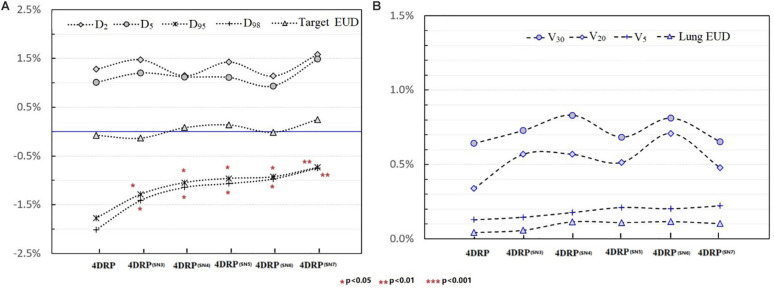
**(A)** The tendency of interplay effect index on DVH metrics of target and **(B)** shows the tendency of interplay effect index of OAR.

Figure 4 shows the static interplay effect index in the target and lung DVH metrics. The interplay effect index $\Delta \,{\text{I}}_{D_{98}}^{\text{CTV}}$ and $\Delta \,{\text{I}}_{D_{95}}^{\text{CTV}}$ decreased as the number of layers increased (Figure 4A). Specifically, the mean values of $\Delta \,{\text{I}}_{D_{98}}^{\text{CTV}}$ were 2.01, 1.48, 1.21, 1.03, 1.01, and 0.92% and the mean values of $\Delta \,{\text{I}}_{D_{95}}^{\text{CTV}}$ were 1.77, 1.42, 1.13, 1.01, 0.91, and 0.89% for 4DRP(**SN3**), 4DRP(**SN4**), 4DRP(**SN5**), 4DRP(**SN6**), and 4DRP(**SN7**), respectively. Compared to $\Delta \,{\text{I}}_{D_{95}}^{\text{CTV}}$ and $\Delta \,{\text{I}}_{D_{98}}^{\text{CTV}}$ in 4DRP, 4DRP(**SN3**) was lower, with a significant difference (*p* < 0.05), whereas no significant differences in the other metrics were observed in the target. In the normal tissue lung, as shown in Figure 4B, the interplay effect index $\Delta \,{\text{I}}_{V_{5}}^{\text{Lung}}$ and $\Delta \,{\text{I}}_{V_{20}}^{\text{Lung}}$ increased when three layers repainting was conducted on 4DRP. The average value was 3% for 4DRP vs. 4.5% for 4DRP(**SN3**) in $\Delta \,{\text{I}}_{V_{5}}^{\text{Lung}}$ and 3.4% for 4DRP vs. 5.1% for 4DRP(**SN3**) in $\Delta \,{\text{I}}_{V_{20}}^{\text{Lung}}$, but no significant difference was observed when the number of iso-energy layers was more than three.

### Relationship Between the Optimal Interplay Effect Mitigation and Target Range of Motion

**FIGURE 5:**
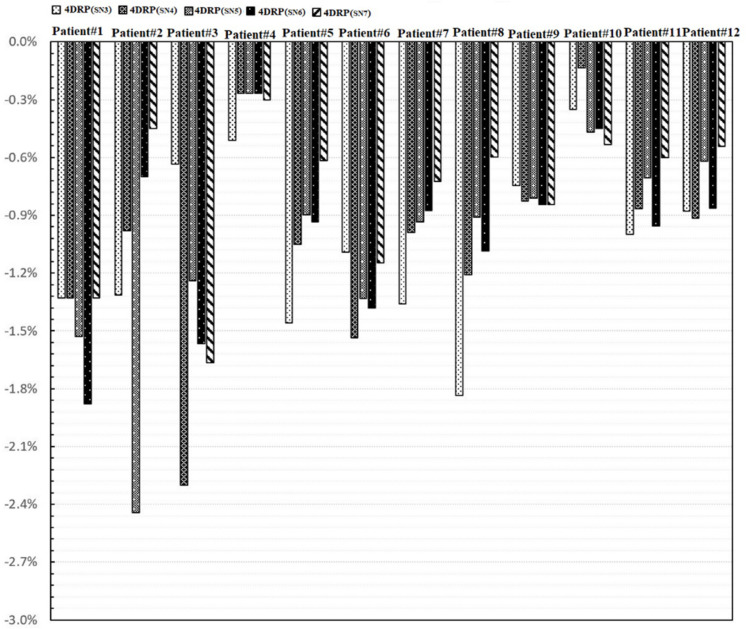
The process of exploring optimal interplay effect mitigation through multiple iso-energy layer rescanning by comparing the interplay index $\Delta \,{\text{I}}_{{\text{D}}_{95}}^{\text{CTV}}$.

**FIGURE 6:**
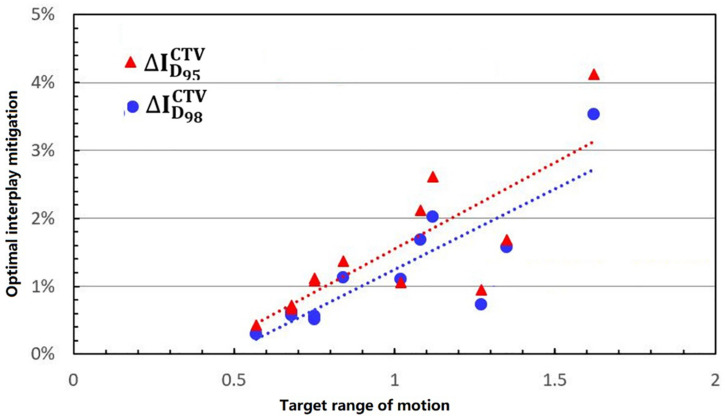
The linear relationship between the optimal interplay effect mitigation on target (D95, D98) and motion range.

Figure 5 shows the process of exploring optimal interplay effect mitigation via multiple iso-energy layer repainting for target coverage metrics. The optimal interplay effect mitigation was attained by comparing the $\Delta \,{\text{I}}_{D_{95}}^{\text{CTV}}$ index among 4DRP(**SN3**), 4DRP(**SN4**), 4DRP(**SN5**), 4DRP(**SN6**), and 4DRP(**SN7**) with the smallest absolute value. Then, a statistical correlation study was conducted to evaluate the correlation between the optimal interplay mitigation and target range of motion that showed a linear relationship between the two (Figure 6). For $\Delta \,{\text{I}}_{D_{98}}^{\text{CTV}}$, the expression was $\Delta {\text{I}}_{D_{98}}^{\text{CTV}}=2.25DVF-0.75,\left(R^{2}=0.63\right)$, while for $\Delta \,{\text{I}}_{D_{95}}^{\text{CTV}}$, it was $\Delta {\text{I}}_{95}^{\text{CTV}}=2.21DVF-1.12,\left(R^{2}=0.73\right)$, where DVF is the maximum deformation vector field or target range of motion. A positive correlation was observed between the optimal interplay effect index and target range of motion.

## Discussion

4D Robust optimization has been proven to mitigate the interplay effects of IMPT planning and can make the difference between the planned dose and delivery dose less than the established criterion (for example, 3%) (18). However, compared to conventional IMPT planning, proton-based SBRT has led to more consideration of the interplay effects and should have strict standards (19). Therefore, it might be ineffective to rely only on 4D Robust optimization alone to mitigate the interplay effects. Thus, we proposed 4D Robust optimization combined with an iso-energy layer repainting strategy in early-stage NSCLC patients.

In the current study, 4D robust plans were evaluated based on the quantitative interplay index for patients with early-stage NSCLC. The results showed that the target coverage decreased due to the interplay effects (Figures 2, 3). Multiple iso-energy layer repainting was subsequently used to mitigate the interplay effects. The target coverage increased as the number of iso-energy repainting layers increased (Figure 4), but this was not applicable for specific patients. Therefore, the optimal iso-energy repainting times were explored based on the quantitative interplay index. A positive linear relationship occurred between the optimal interplay effect mitigation and target range of motion. Our results confirmed that 4D Robust optimization combined with iso-energy layer repainting technology further mitigated the interplay effects, which has important clinical significance.

The motion of lung tumors was evaluated based on 4DCT images. Only 35 to 39% of the tumors moved more than 5 mm in locally advanced NSCLC, but the percentage increased to 50% for early-stage NSCLC (5). In the current study, the target range of motion was assessed using the maximum DVLs of the DIR within the target. The results showed that all of the patients had a more than 5-mm range of motion, which was also evaluated by the difference between the ITV volume and mean CTV volume over all phases (the CTV change rate in the 4DCT images). Figure 6 shows that there was a positive linear relationship between the tumor range of motion and CTV change rate. Thus, the target range of motion was obtained based on the differences in the ITV and CTV contours in the 4DCT images. DIR errors should be considered. Anaconda, implemented in RayStation, is a hybrid method utilizing a combination of image intensities and controlling structures from contoured image sets (13). Anaconda demonstrates a good performance in the thoracic region compared to other commercially available algorithms based on previous studies (14).

In practice, when using spot scanning, the number of spot rescans should be proportional to the patients’ spot weights in a range of motion up to 5 mm. However, the optimal number of repainting layers remains debatable due to patient- and machine-specific parameters, such as the patient breathing cycle, energy switch times, and other factors (20). Seco et al. (21) investigated a phase-controlled repainting and breathing-sampled strategy in which the number of rescans was decided using a motion-monitoring system. Engwall et al. (9) investigated offline breath-sampled layered repainting methods in which the number of iso-energy layers was spread uniformly throughout the breathing cycle, the optimal method of mitigating interplay effects. In the current study, multiple iso-energy layer repainting times were used to explore the optimal number of interplay mitigation rescans based on the quantitative metrics (Figure 5). A positive linear correlation occurred between the optimal interplay effect mitigation and target range of motion, demonstrating that breathing motions are dominated by the interplay effect.

One limitation of this study was associated with the method of simply dividing each layer in the present number of layers, which resulted in small weighted spots that might have been deleted after multiple iso-energy rescans. The dose distribution before and after the iso-energy rescans was compared to avoid this scenario in this study.

## Conclusion

In proton-based SBRT, interplay effects degrade the target dose distribution and can be mitigated using iso-energy layer repainting techniques. However, in this study, there was no significant correlation between the number of repainting layers and improvement in the dose distributions. We recommend using the optimal layer repainting times based on the interplay effect index according to the patient characteristics.

## Data Availability Statement

The raw data supporting the conclusion of this article will be made available by the authors, without undue reservation.

## Ethics Statement

Ethics approval for this study was obtained from the Chongqing University Cancer Hospital’s Ethics Committee. All patients gave written informed consent, and all methods were performed in accordance with the relevant guidelines and regulations.

## Author Contributions

HS conceived and designed the study, and collected and sorted the data. LW and HS wrote the manuscript. LW carried out the experiments, data analysis, and statistical analysis with guidance from HS. FJ and YW assisted with statistical analysis. All authors edited the manuscript, and critically reviewed and approved the manuscript.

## Conflict of Interest

HS was employed by the company RaySearch. The remaining authors declare that the research was conducted in the absence of any commercial or financial relationships that could be construed as a potential conflict of interest.

## Footnotes

1 https://www.microsoft.com

## Abbreviations

4DDD: 4D dynamic dose
4DRP: 4D robust optimization plans
4DSD: 4D static dose
CTVs: clinical target volumes
DIR: deformable image registration
DVF: deformation vector field
IMPT: intensity-modulated proton therapy
ITVs: internal target volumes
NSCLC: non-small cell lung cancer
OARs: organs at risk
SBRT: stereotactic body radiotherapy.
